# Beyond Culture and Language: Access to Diabetes Preventive Health Services among Somali Women in Norway

**DOI:** 10.1155/2015/549795

**Published:** 2015-07-22

**Authors:** Abdi A. Gele, Liv Elin Torheim, Kjell Sverre Pettersen, Bernadette Kumar

**Affiliations:** ^1^Department of Nursing and Health Promotion, Faculty of Health Sciences, Oslo and Akershus University College of Applied Sciences, P.O. Box 4, Olavs Plass Street, 0130 Oslo, Norway; ^2^Norwegian Centre for Minority Health Research, P.O. Box 4956, Nydalen, 0424 Oslo, Norway

## Abstract

Despite the high prevalence of type 2 diabetes in some immigrant and refugee communities in Norway, there is very little information available on their utilization of diabetes prevention interventions, particularly for women from Somali immigrant communities. A qualitative study of 30 Somali immigrant women aged 25 years and over was carried out in the Oslo area. Unstructured interviews were used to explore women's knowledge of diabetes, their access to preventive health facilities, and factors impeding their reception of preventive health programs targeted for the prevention of type 2 diabetes. The study participants were found to have a good knowledge of diabetes. They knew that a sedentary lifestyle and unhealthy diet are among the risk factors for diabetes. Regardless of their knowledge, participants reported a sedentary lifestyle accompanied with the consumption of an unhealthy diet. This was attributed to a lack of access to tailored physical activity services and poor access to health information. Considering gender-exclusive training facilities for Somali immigrant women and others with similar needs, in addition to access to tailored health information on diet, may encourage Somali women to adopt a healthy lifestyle, and it will definitely contribute to a national strategy for the prevention of diabetes.

## 1. Background

Type 2 diabetes mellitus (T2D) represents a growing threat to human health given the explosion of its incidence in the last few years. According to the International Diabetes Federation (IDF), 8.3% of the world's adults (382 million people) have diabetes, and the number is set to rise beyond 592 million within the next two decades [1]. In Europe alone, over 32 million people live with T2D, and approximately a further six million are unaware that they are living with it [2]. Ethnic minority populations in Europe have been particularly affected by T2D compared to the population of European origin [3–6]. Not only do migrant groups develop T2D at a younger age, they also have a higher morbidity and mortality from T2D and related complications [3, 4, 7]. The available data indicates that, in particular, African immigrants experience a high burden of diabetes [8–11]. A premigration self-reported BMI of 23.4 kg/m^2^ and postmigration BMI of 27.4 kg/m^2^ were found among Ghanaian women in Australia [10]. The same study found a prevalence of type 2 diabetes of 11% in Ghanaian women, with only 23% of those affected being aware of their diabetes status, thereby indicating the lack of an awareness of diabetes among African immigrant women [10]. A similar situation was reported from the African Diaspora of West African decedents [8, 9], as well as different African communities in Israel, Europe, and the US [11]. Somali women are of particular concern given their unique vulnerability in developing T2D. Somali women are almost exclusively Muslim, and their religious restrictions, family, and migration-related issues might become barriers to exercise. Muslims require that women wear loose-fitting clothing that covers the body from head to ankle, particularly when in the presence of males [12]. Consequently, prior study found a high proportion of physical inactivity among Somali women [13]. Moreover, Somali women had had a variety of traumatic experiences in their home countries, while they face a number of challenges when adapting to their new country such as language barriers, discrimination, poor understanding of country's health system, and religious differences. Consequently, an increase in sedentary behavior, coupled with a drastic change in diet among Somali women, may lead to cardiovascular risk, overweight, and diabetes [14]. Adequate actions for the prevention of T2D among Somali refugees women are imperative, given the size of this community in Europe and their vulnerability to T2D.

Norway's immigrant population (immigrants and Norwegians born to immigrant parents) rose from 183,000 persons (4.3%) in 1992 to 759,000 persons (14.9%) in 2014 [15]. Accordingly, a growing body of research has begun to look at the health of Norway's immigrant populations [16–18]. Studies have demonstrated that immigrants have a tendency to adopt a Western lifestyle, including an increase in the adoption of poorer eating habits and an increase in a sedentary lifestyle [17, 19, 20]. Immigration to the West is usually accompanied by environmental and lifestyle changes that can increase immigrant's risk for diabetes. A substantial amount of the literature in Sweden, Denmark, and Norway presented extremely higher prevalence rates of T2D among immigrants than nonimmigrants [17, 19, 20]. However, these disparities are heterogeneous among immigrants, with refugees possibly being more susceptible to factors that fuel health disparities. Refugees experience family and social disruption and a subsequent altered health, but this is even greater when associated with civil conflict and homeland violence [21]. A study of diabetes among patients from four countries that have undergone civil war found the highest prevalence of diabetes (32%) among Somalis [22]. The same study found a prevalence of obesity of 41.1% among Somalis compared to 8.6% and 19.5% among Vietnamese and Cambodians, respectively. A high level of physical inactivity and a higher BMI were also observed among Somalis in Norway, the UK, and New Zealand [13, 23, 24].

Over the last few decades many studies have demonstrated that overweight/obesity and the underlying energy-balance-related behaviors; in particular, diet and physical activity are important modifiable risk factors for T2D [25, 26]. Efficacy trials that evaluated the potential of this approach have consistently shown that T2D can be prevented through weight reduction and behavioral changes related to healthy nutrition and an adequate amount of physical activity [27, 28]. However, transferring such evidence into effective community prevention programs requires an understanding of the specific needs of these communities prior to the application of any type of intervention [29]. Accordingly, the public health paradigm is currently shifting to accommodate innovative approaches for understanding community perspectives [30]. The aim of this study is therefore to explore the experiences of Somali immigrant women in the reception of preventive health services in relation to T2D.

## 2. African Immigrants in Norway

The population of African-born immigrants in Norway is estimated to be nearly 97,152, with people of Somali descent representing nearly 50% of them [15]. The majority of Somali immigrants came to Norway after 2002 as refugees, with almost half living in Oslo [15]. Somalis are primarily refugees with the experience of decades of civil conflict that can increase their risk to noncommunicable diseases [31]. Nonetheless, immigrant health needs in Norway are frequently reported in general, which can mask important distinctions in health needs for specific groups [32]. While explicit data on ethnicity is becoming increasingly important for health providers, the available literature in Norway underrepresents African immigrants, with this disparity leading to a limited amount of information about the health needs of this particularly vulnerable group [32].

The African community in Norway stands out as the worst off by any socioeconomic standard compared to other immigrants. For instance, the employment rate of Somalis in Norway was 25.8% in 2001 compared to 45% among Pakistanis [33]. While there is literature on preventive health care among many minority ethnic groups, research addressing the health of African immigrant women is sparse, and most of the few available studies on this group address reproductive health [34–37]. The absence of research on preventive health utilization among Somali women is of particular concern because Somalis are suspected to be unfamiliar with the principles of preventive health care in the West [38]. Studies reported significant discrepancies in the receipt of preventive health measures between Somali immigrants and non-Somali ethnic groups [38]. Largely shaped by a lifelong deprivation of quality health systems in their home country, the exposure to war, and the experience of refugee life, Somalis were reported to focus on issues of immediate survival, with many of them having no reference for the notion of prevention and the long-term management of chronic diseases [38]. In the same vein, consulting with a general practitioner (GP) for chronic diseases screening and illness prevention is unfamiliar to most Somalis, who are accustomed to only seeking health care when ill [39]. Consequently, most chronic diseases are likely to go undetected until complications arise, with the reason for this being that no disease is perceived as a problem unless it is accompanied by symptoms that drive one to seek health care and that warrant treatment such as fever, diarrhea, pain, and cough [40]. The utilization of preventive health measures is not driven by any immediate health needs because cardiovascular risk factors such as sedentary lifestyle and unhealthy eating do not rise to the level of being an immediately life threatening or anxiety elevating condition. It is this nature of engaging in preventive health measures that has raised special interest in the extent to which Somali immigrant women use preventive health care in regard to diabetes.

Culturally tailored and language-specific educational programs for preventive health measures are more likely to engage immigrants and result in more efficacious outcomes if they are well designed [41]. The Norwegian diabetes strategy recognizes the significant disparities in knowledge on healthy eating and physical activity among communities, while reducing this disparity is seen as an important goal in the prevention of diabetes [42]. Consequently, structural mechanisms involving activity-promoting residential areas and the motivation for active lifestyle and healthy eating through robust guidance, as well as population-based information and attitude-shaping work, were prioritized in the national diabetes strategy [42]. Furthermore, the existence of particularly vulnerable groups with special facilitation needs who are not easily identified by the general population-based and structural mechanisms was noted in the strategy. Despite this, the preventive health needs of Norway's growing and diverse immigrant communities were understated in the national diabetes strategy document. This study's overarching goals are to explore Somali women's knowledge on diabetes and its risk factors and access to preventive health information with regard to diabetes and factors impeding the reception of preventive health programs among this community.

## 3. Methodology

### 3.1. Study Design

A qualitative study using unstructured interviews was conducted in Oslo from August to November of 2014. An unstructured interview is not only a flexible tool for assessing people's experiences and their attitudes and feelings of reality, but one that does not impose any a priori categorization that might limit the field of inquiry [33]. Gaining trust and establishing good relationships are essential to the success of unstructured interviews, as only when a trustful and harmonious relationship is cultivated can the interviewee share his/her knowledge and experience on topics of interest to the interviewer.

A snowball sampling of 30 Somali women aged ≥25, all of them being first generation immigrant, was conducted in Oslo. We followed common research ethics principles in the carrying out of this study, including informed consent, where the right to refuse, as well as withdrawal and confidentiality, was explained to each participant. Afterwards, verbal consent was obtained from the participants, and this study was ethically cleared by the National Data Registry of Norway.

The first author who is a Somali ethnic carried out the interviews, together with an assistant. The interviewers developed trust and an intimate relationship with participants prior to initiation of the interviews. Moreover, participants were given information about the study's objectives, as well as the research question that the study intended to answer prior to the interviews. The interviews were conducted in the Somali language, which was the native language of both the participants and the interviewers.

### 3.2. Content of the Interview

During the interviews, the terms* ka hortagga* (prevention),* sonkorta*, or* macaanka* (diabetes) were used, and the participants were asked about their understanding of diabetes, in addition to their perspectives regarding the prevention of the disease. We explored their access to preventive health information and preventive health services, and the factors that prevent Somali women from utilizing existing preventive services and receipt of health information were also examined. Lastly, the acceptable means of delivering preventive health information and preventive health services were explored through women's perspectives. The interview process continued until it was clear that no new information was emerging from the additional interviews, that is, when saturation was achieved.

### 3.3. Analysis

The research assistant transcribed the interviews verbatim, and the transcripts were thoroughly read several times by the first author and a research assistant. Thematic analysis was used to identify and analyze important themes [35], with the coding process involving recognizing and encoding the identified themes prior to interpretation [36]. According to Leininger [37], themes can be identified by bringing together fragments of ideas, experiences, and beliefs that are often meaningless when viewed alone. For that reason, themes that emerged from the informants' stories were pieced together to form a comprehensive picture of the participants' shared experience [38]. The themes that were identified through coding were divided into categories based on the participants' experience in preventive health utilization with regard to diabetes [39]. We employed a multimethod design in this study, and the consistency of the findings from different methods we used (i.e., interviews and quantitative data which were not yet published) has served to ensure the trustworthiness and credibility of the study's results. Furthermore, two study participants who spoke English have read the final paper and verified that what they said is presented correctly in the paper.

## 4. Results

The study explored Somali women's knowledge on diabetes and their access to health information, as well as factors impeding their access to preventive health services. The participants' responses to most of the questions were similar regardless of the number of years of their stay in Norway; as a result, their responses were thematically categorized into the following six topics.

### 4.1. Knowledge on Diabetes

There was a great variability in diabetes knowledge among the participants. However, the majority of participants considered diabetes to be a dangerous but preventable disease. While some participants thought that diabetes was caused by a consumption of excess sugar and that it can be prevented by reducing sugar consumption, others demonstrated a high level of understanding about the prevention methods of diabetes. In the Somali language, there is an overlap in relation to the terms “risk” and “cause”; thus it is hard for many Somalis to differentiate risks from causes. Therefore, most participants used causes rather than risks as they discussed various risk factors for diabetes. By answering a question about what they knew about the risks, symptoms, and prevention of diabetes, some participants stated the following:

*Diabetes affects all ages. I don't know about this disease that much. There are two types, 1 and 2. It can be caused by the consumption of an unhealthy diet and being sedentary (Participant  no.  3).*

*I think the causes include stress, an unbalanced diet and a sedentary lifestyle. For Somalis, it is often related to stress (Participant  no.  2).*

*Diabetes is caused by excess weight, stress and the use of lots of sugar. It can be controlled through physical activity and diet control (Participant  no.  8).*

*Diabetes is a dangerous disease that requires training and the avoidance of all habits that can increase body sugars (Participant  no.  26).*

*I have heard about diabetes and it has symptoms like shaking, tiredness, headache and excess urination. The type 2 diabetes is dangerous. It can be managed if someone works on his diet and does physical activities and reduces their weight (Participant  no.  16).*

Some participants demonstrated a good knowledge on diabetes because they either had close family members with diabetes, or they were affected by the sad consequence of diabetes through the death of parents or other family members:

*I know diabetes because both my mom and dad have diabetes. I know it is a dangerous disease (Participant  no.   25).*

*My mom has diabetes and my grandmother died from it. I have heard it is a dangerous disease, and the symptoms include burning, excessive urination, and it can also lead to kidney failure and amputations. There is no treatment that can cures diabetes (Participant  no.  21).*

Despite the fact that most participants knew the risk factors for diabetes, its symptoms, and preventive measures, some participants indicated that they did not receive this information from any official channel such as GPs and diabetes association, but instead through family members and networks:

*I did not get detailed information about diabetes prevention except that people should go for training. I have never heard about it from my doctor, but I heard it from people. I was diagnosed with diabetes while I was pregnant and later after the delivery I was told I am diabetes free (Participant  no.  25).*

Because Somalis call diabetes* Sonkor*, which means* sugar* in English, some participants reported that diabetes is the result of a consumption of excess sugar, and thus reducing their sugar consumption may be enough to prevent the disease:

*I know people who have the disease here and in Somalia. I think it is caused by using too much sugar (Participant  no.  15).*

*I know diabetes is a disease that affects people if they consume too much sugar. It can be prevented through physical activity (Participant  no.  29).*

*I think people are not aware about the connection between their food consumption and diabetes. For us Somalis, we associate diabetes with consumption of sugar. If people reduce the amount of sugar consumed, they think it is done. They never associate other types of food with diabetes. They tell you that they do not use that much sugar in the tea or coffee, so they are not at risk for diabetes (Participant  no.  7).*

### 4.2. Diet Preferences

Many participants associated an unhealthy diet with diabetes. Accordingly, they were asked about their habitual diet. With few exceptions, the majority of participants agreed that the main components of their diet were Injera bread, rice, pasta, meat, and tea or coffee with lots of sugar. While the majority was aware that their diet was not healthy, they did not express any intention of changing their dietary habits:

*I eat everything that comes into my sight without classification. I drink lots of tea, I cannot drink tea without sugar, so I use lots of sugar because I love it (Participant  no.  6).*

*I eat Somali food, I also eat bread, I drink tea with lots of sugar, I can't stop that (Participant  no.  1).*

*When we meet we always make tea with lots of sugar and eat sweets like Xalwo. We love it and we can hardly stop it (Participant  no.  5).*

One of the participants reported that they usually consume a traditional Somali diet, but when they receive health information they temporarily reconsider their diet. However, when there is no health information, they indicated that they stick to their traditional diet:

*I didn't change my lifestyle, I just use traditional foods. I may stop temporarily when I read information that scares me (Participant  no.  22).*

One participant indicated a generational difference in food preference between old people and the young people who grew up in Norway. The younger generation may be more concerned about their health and diet, while the older generation who came to Norway as adults do not know about the concept of a healthy diet:

*I don't believe that all Somalis use Somali food. Young people who studied in Norway know about healthy food and they use it, they go to exercise classes, but Somali mothers may not have that information. They may not change food because they do not have information about the risks associated with their lifestyle (Participant  no.  28).*

By answering a question on why they use foods such as pasta and rice with meat soup and tea with lots of sugar, most participants stressed their knowledge of the unhealthy nature of their diet, though they indicated that they do not intend to modify their unhealthy diet. One participant also indicated her low consumption of fruits and vegetables:

*I don't drink tea without half of the cup being filled with sugar. I love the tea. We cannot get rid of our traditional food because this is what we are used to. But the bad thing is that we don't like training too, so food with lots of sugar and fats plus a sedentary lifestyle is a problem. I think most people know about the risk associated with our food but we cannot stop it. If I try to eat vegetable and fruits and fish, I may not enjoy it, I eat the food that I enjoy regardless of whether it is healthy or unhealthy (Participant  no.  29).*

Some participants demonstrated that the problem is not their traditional diet, but when this type of food is accompanied with a sedentary lifestyle. The dominant perception among the participants was the fact that they were physically active in their home countries because they had a climate that was supportive for an active lifestyle. However, they become sedentary when they come to Norway because of an unfavorable climate that has limited their movement:

*I eat Somali food, but it was healthy in Somalia because I used to walk a lot. Here my weight has increased because I eat food and I am sitting the whole day (Participant  no.  13).*

*Every community has a traditional food. It is our tradition to use rice, pasta and tea with lots of sugar. However, information is important. Norway is a cold country where we experience an unhealthy lifestyle. Our country (Somalia) is a warm country where people can walk and exercise (Participant  no.  23).*

### 4.3. Barriers to Being Physically Active

While most participants had a good understanding about the importance of a healthy diet and physical activity in the prevention of diabetes, some participants attributed the hurdles that Somali women face in Norway, with regard to physical activity, to the absence of culturally sensitive structures. While almost all of the participants were aware of the importance of physical activity, they believed that the available structures are not in line with their needs:

*Most of the women would love to go for training. But there are barriers. They have huge responsibilities. Women have small children and when the children go to a school or nursery, mothers need to go to school or a job training course. A lack of time and financial barriers are there, but people do care about their health because it is important for them. Gyms are expensive and if a mother does not have a job, she cannot pay 500 kroner every month. If someone has the financial capability, he/she can hire a housemaid (to  take  care  of  the  children) for two hours and go to do some physical activity. But most of us have several children, and we don't have paid jobs, only unpaid training jobs, while others go to schools, so there are both time and financial barriers (Participant  no.  25).*

While time and financial accessibility may be important barriers for physical activity among Somali women, several women indicated that the available training facilities are not gender exclusive, which collides with their culture:

*We Somali women have a number of problems in going to do physical activity, such as at a gym. You meet men in the gyms and they look at you. This is not what we are used to. I came to Norway as an adult. I cannot change my culture, so I cannot force myself to use those gyms, nor do I have a training facility at home, so I do not have many options, only one option I have is to sit home and hope the best for my health (Participant  no.  13).*

One participant indicated that Somali women were not used to exposing their body to outsiders, and, by doing so, they may feel shame and stigma. Still, they feel comfortable among themselves. Another participant used the example of a facility that is gender-specific that Somali women use often and indicated that if such facilities were made available to them, they would get motivated to be physically active:

*There are certain places where women can swim alone. I went to the Furuset swimming center two weeks ago and there were many Somali women who were participated in it. It is provided by the Mira center and they offer it once every two weeks. I think if such services are made available and accessible to women they would use it (Participant  no.  28).*

*We should be given access to facilities where we can exercise without feeling shame and the fear of stigma from other people (Participant  no.  8).*

Traumatized by the conflicts back home and a subsequent hazardous migration route characterized by rape, murder, and other types of violence against women, some participants demonstrated a feeling of insecurity towards the available training facilities:

*There was a day I went to a gym. That was the day I decided not to go to a gym again. It was on a weekend, and there was nobody in the gym, I THOUGHT if a strong man comes in and rapes me then what can I do. I became scared and ran away from the gym. I have heard about a lot of violence against women from the news. I think women should find out other alternatives to be active. For example, physical activity is important for our health so women should go to gyms in groups but not alone. If that is not possible women should buy equipment and exercise in their homes. They can go on long walks, etc. (Participant  no.  15).*

### 4.4. Requirement of Culturally Sensitive Physical Activity Facilities

Several participants repeatedly mentioned that they sometimes use a far-away swimming facility that is for women only and to which they go some few days every month. They repeatedly mentioned that access to such a tailored training facility might motivate them to be more physically active:

*There is uni-gender swimming pool in Furuset, and most Somali women go there to swim. Some of the women come from neighborhoods that are far away. If such a facility was made more accessible, women would get motivated to do more physical activity (Participant  no.  18).*

*The best solution is the provision of training facilities that are specific to women. I believe if such facilities were made available within our reach and at a reasonable price, I am sure Somali women would use it and diabetes could be prevented. Sometimes we pay money to gyms, but we drop it when we find that men and women are exercising together (Participant  no.  12).*

### 4.5. Need for Preventive Information and Tailored Structure

As frequently mentioned by earlier respondents, Somali women reported that they did not receive any preventive health information or information about available facilities and services for disease prevention. However, some participants reported that health information alone may not be enough for the prevention of diseases in the absence of culturally sensitive structure:

*There are telephone numbers to call for cancer information, but information alone doesn't help. People need facilities to use. When people receive information, if there is no facility that fits them, information alone cannot be helpful (Participant  no.  20).*

*People do not receive instructions of where the information can be found. They may not receive information until they become sick, and by that time the information is less relevant. We do not have information about diseases (Participant  no.  13).*

### 4.6. Preferred Means for Preventive Health Delivery

Participants were asked about the preferable means of health information delivery to Somali women in Norway. They overwhelmingly suggested receiving information in their own language and through oral information as opposed to written information. One participant elaborated about other successful intervention campaigns that were delivered to them, and she suggested that similar methods can be used in the prevention of other diseases. According to most of the participants, information campaigns should be accompanied by culturally, geographically, and financially accessible physical activity facilities:

*People can be given courses about diabetes prevention, and there are immigrant organizations that can reach Somalis. For example, there is the practice of FGM, I think you know that FGM information is given to every one of us, as the campaigns against FGM have reached out to everyone. If similar channels were used to deliver information about diabetes, I think people could improve their health. It is just matter of allowing people to understand the risk they are undergoing. Then they will change their lifestyle (Participant  no.  16).*

*Information can be provided through campaigns that highlight the importance of physical activities on health. It can also be delivered through brochures and through speeches, exactly as we speak about it now. We need to get information in our language (Participant  no.  26).*

## 5. Discussion

This study explored the utilization of diabetes prevention interventions and access to health information among Somali immigrant women in Oslo, a topic that has not previously been documented in the literature. The results show a high awareness of diabetes, its risk factors (a sedentary lifestyle and unhealthy diet), and its prevention among the study participants. Despite having a good knowledge about diabetes, most participants reported that they are not only at risk for diabetes but, given their unhealthy lifestyle, they are also incapable of avoiding it. An earlier study noted that the disparities in the utilization of preventive health services are greater for Somali women compared to other communities [38]. The health beliefs model assumes that health behavior is influenced by five factors: perceived susceptibility to a particular illness; perceived severity of the illness; perceived benefits in changing one's behavior; and perceived barrier to changing one's behavior and cues to action [43]. Among these factors, perceived barriers are the strongest predictor of whether or not an individual will adopt a healthy behavior [43]. In the present study, Somali women's unhealthy lifestyle was largely attributed to the absence of a tailored structure, as opposed to a lack of awareness of the benefits of physical activity. This result is in accordance with the recent finding in Sweden that Somali women are aware of the benefits of physical activity [44]. Access to tailored prevention intervention may motivate Somali women toward engaging in optimal health practices, with a tailored intervention implying the development of health messages and facilities that are consistent with the characteristics, needs, and cultural beliefs of a given community [43]. In the absence of a tailored intervention that motivates a healthy lifestyle, women's knowledge and attitude towards physical activity and a healthy diet (perceived benefit) may be of little help.

Our results highlight the challenges that Somali women face in becoming physically active, which include time pressure, a lack of financial affordability for training facilities, and an absence of a tailored physical activity environment. Norway is a welfare state with a very generous health and social system that attracts migration; however, while many people see the affordability of health care as less relevant in Norway, refugees and immigrants may be facing affordability problems in gaining access to preventive health care. This finding concurs with prior studies that report time pressure [44] and a lack of resources [45] as major barriers to the utilization of available physical activity services among Somali immigrants women. While this study reports that mixed-gender training facilities may be a major issue that limits accessibility to physical activity among Somali women, studies found that gender-exclusive facilities help overcome this barrier [46]. Participation in physical activity is a woman's human right that is recognized by both the Convention of Elimination of Discrimination against Women (CEDAW) and the International Charter of Physical Education and Sport (UNESCO, 1978) [47]. Similarly, Islam recognizes the importance of physical activity among women. Nonetheless, studies noted that dress codes and gender separations are areas where Western sports ideology differs from the Islamic framework of physical activity [47]. When such differences are not taken into account in relation to immigrant's health, subjects may choose to either compromise their beliefs and utilize the available facilities or avoid using facilities and remain sedentary [47]. The need for gender-exclusive facilities was reported to extend beyond Somali communities to other Muslims, East Africans, and others with similar preferences [46]. Considering gender-exclusive training facilities for Somali immigrant women and others with similar needs may encourage them to be physically active, and it will also contribute to the national strategy for the prevention of diabetes.

Study participants demonstrated a worry about an unhealthy diet accompanied by a sedentary lifestyle. Our study echoed the results in the UK, indicating that the typical diet of Somali immigrants largely consisted of rice, pasta, and red meat, with a low consumption of fruit and vegetables [48]. It is a diet that, when combined with a more sedentary lifestyle than they had in Africa, can lead straight to diabetes [40]. A cultural association between the consumption of fruits/vegetables and poverty and between red meat and affluence has been reported from Somalis [14]. Consequently, an earlier study that investigated the dietary habits of the Somali population in Liverpool (which has had a long-established Somali community since the 1960s) recommended that Somali immigrants' traditional eating habits needed some modification [13]. However, studies have found that immigrants are aware of the need to change their dietary behavior, but they lack both information and motivation [49], and our study reports a similar finding. A previous study that explored the role of ethnic identity in maintaining health behavior among Southeast Asian immigrants found a strong association between ethnic identity and maintaining a traditional diet over time. The study suggested that programs that promote cultural retention may increase the success of health nutrition counselling for this community [50].

The food choices of the Somali immigrant women in Oslo might have influenced by a series of debilities such as alienation, lack of education, lack of nutritional knowledge, and motivation. While lack of information and knowledge about foods and nutrient contents among study participants might play a part, motivation to change is likely to be much more important. Food choice is often influenced by many interrelating factors, including various physiological, social, and cultural factors [51], and these need to be taken into account when considering dietary interventions among Somali immigrant women. The lack of motivation to change, which was reported by some participants, may be related to optimistic bias, where people underestimate the risk to themselves relative to others, and this factor might lead people to take less note of health education messages. There may be an issue of moral concern too, which may be important for the choice of unfamiliar foods and for foods to be eaten by others. An awareness of the association between an unhealthy diet and diabetes is vital for Somali women in Norway, as is increasing their knowledge on the availability of a healthy diet that they may not be familiar with.

In addition to a sedentary lifestyle and unhealthy diet, Somali women demonstrated a lack of health information about the preventive health services available to them, in terms of what services can be found and Norway's diabetes prevention strategy as a whole. In Norway, most of the available health information is likely to be based on cultural homogeneity and universal preferences for independent learning. Thus, the health information is often delivered through brochures, materials available online, posters, booklets, the internet, and media-based announcements. These materials are sometimes available in the Somali language, assuming a high level of health literacy among Somalis. Even so, Somali women were reported to be unfamiliar with those services [52]. Most respondents in this study preferred information in the Somali language and through oral communication, plus visual learning such as pictures. This finding concurs with the finding of a prior study among Somali women in the US, which suggested in-person discussions as a suitable information delivery method for Somali women [41]. One of the most successful health promotion approaches among immigrant women includes the use of lay leaders (trained individuals from ethnocultural groups) to help increase the relevancy of health messages and to reduce perceived barriers to behavioral change [53, 54].

The study has limitations. First, the reported result is the perspectives of 30 participants, but it is unclear whether this is the view of the majority of Somali women in Norway. Moreover, the possible role of stress in forming attitudes to diabetes was not further explored during the interview even though some participants had reported their perception of it. Moreover, we did not explore participants' perception on leisure-time activities such as walking or jogging. However, traditional Somali life never involves leisure-time physical activity; thus, one cannot expect to compensate for the low daily activity level with leisure-time activity [44]. Despite the limitations, this study identified the preventive health needs, as well as the barriers and facilitators in gaining access to and the utilization of preventive health services of a group at high risk of developing T2D. The study found that a nexus of social, structural, cultural, and economic barriers creates a pressure that makes it difficult for Somali women to utilize diabetes preventive health services. The findings may be used for the implementation of some specific health promotion programs, in addition to the establishment of tailored physical activity facilities, and nutrition training for Somali women, which would fill the gaps in their preventive health access and utilization.
